# The morphological classification and clinical significance of atlas vertebral artery sulcus based on computed tomography three-dimensional reconstruction

**DOI:** 10.1007/s00276-023-03079-x

**Published:** 2023-01-30

**Authors:** Dingxiang Hu, Changhui Li, Liang Chen, Chenxi Ma, He Huang, Ruiqing Zheng

**Affiliations:** 1 Department of Orthopedics, Luxian People’s Hospital, Sichuan, 646100 China; 2 Department of Radiology, Luxian People’s Hospital, Sichuan, 646100 China; 3 Recovery Units, Luxian People’s Hospital, Sichuan, 646100 China

**Keywords:** Atlas, Vertebral artery sulcus, Anatomy, Classification, Three-dimensional reconstruction

## Abstract

**Objective:**

The purpose of this study was to research the morphological classification and clinical significance of vertebral artery sulcus on atlas based on CT three-dimensional reconstruction.

**Methods:**

Three-dimensional reconstruction images of 300 adult atlases were collected. A total of 600 atlas vertebral artery sulci were selected in this study. The parameters required for placement of C1 pedicle screw, including depth of grinding drilling (ao), width (cd), length ab), height (H), lateral wall thickness (L1), inner wall thickness (L2), medial angle (∠α), and the cephalad angle to the transverse plane of atlas pedicle (∠β), were measured.

**Results:**

CT three-dimensional reconstruction images showed that there were five types of atlas vertebral artery sulci: no process type (*n* = 494 cases, 82.33%), upper process type (*n* = 29, 4.83%), lower process type (*n* = 25, 4.17%), double process type (*n* = 19, 3.17%), and posterior ring type (33, 5.50%). One-way ANOVA tests showed that the five groups differed significantly in the parameter of ao, L2, H, ∠α and ∠β. One-way ANOVA with the LSD post hoc tests showed that the parameter ao of the group of no process type was less than that of the group of upper or lower process type (*P* < 0.05), and ao of the group of lower process or posterior ring type was less than that of the group of the upper type (*P* < 0.05). The parameter of ao of the male group was larger than that of the female group.

**Conclusion:**

No process type of the atlas vertebral artery sulcus was the most common, and the medial angle and cephalad angle of the atlas pedicle in this type were the smallest. When pedicle screws are inserted, the above two angles should not be too large. Male's ao was larger than that of female's. All these findings should be considered to avoid the deviation of the nail track.

## Introduction

Atlantoaxial vertebra is an important structure connecting the skull and cervical vertebra. Its anatomical position is deep, complex and adjacent to the medulla oblongata, vertebral artery and nerve root. As the segment with the largest activity in the cervical spine, atlantoaxial joints undertake the rotation function of more than 50% of the cervical spine [1]. Atlantoaxial fracture is a common condition in upper cervical spine injury, which is one of the common reasons for atlantoaxial instability [2]. In addition, inflammation, tumor, congenital malformation, degeneration and so on are also common reasons for atlantoaxial instability, which might lead to limb sensory and motor dysfunction due to the adjacent medulla oblongata and cervical spinal cord compression, and even respiratory and circulatory center damage and death of patients [3, 4].

Since atlantoaxial instability has a huge potential risk, active surgical treatment is often used in the clinic. The objective of surgical treatment was to relieve cervical spinal cord compression, restore normal anatomic relationship and reconstruct the stability of the atlas and axis [5]. Because the atlas pedicle screw has better biomechanical stability, it has been recognized by clinical orthopedic surgeons [5, 6]. However, the atlas pedicle structure is relatively complex, and the anatomical variability large. In addition, it is adjacent to the spinal cord, vertebral artery and other tissues, which increase the difficulty of clinical treatment for patients with atlantoaxial instability [7]. Many scholars have proposed different insertion points and angles for the accurate insertion of the atlas pedicle screw, but the probability of damage to the surrounding vascular nerve is still large in practice [8–10]. Some scholars have described the morphology of the atlas vertebral artery sulcus, but there are few reports focusing on the effect of the morphology of the atlas vertebral artery sulcus on pedicle screw placement [11–13]. In this study, we collected CT three-dimensional reconstruction data of the atlas, observed the morphology of the vertebral artery sulcus, analyzed the pedicle parameters, and provided accurate anatomical basis for atlas pedicle screw placement.

## Materials and methods

### Volunteers

From August 2017 to June 2021, 309 volunteers with no or mild cervical spine degeneration were enrolled in this study. This study was approved by the Luxian People's Hospital Review Board (IRB Number, 2021-0701). The inclusion criteria were: healthy adult with complete atlas CT scan data; with normal atlas anatomical structure; no surgical history affecting the atlas. Participants with unclear atlas CT scan data or abnormal anatomical structure of the atlantoaxial vertebra were excluded. According to the inclusion and exclusion criteria, nine cases were excluded, including four cases of atlantoaxial dislocation, two cases of atlas deformity, one case of atlantooccipital fusion, and two cases of atlantoaxial image difficult to clearly reconstruct. The final 300 patients were included in this study (156 male and 144 female) with an average age of 43.22 ± 13.24 years.

### Radiological classification and measurement

Multi-slice spiral CT imaging (Optima Ct 680, GE, USA) was used in this study. The parameters of CT imaging were 120 kV, 110 mA, and the interval with 0.625 mm section. The slice source image obtained from the axial plane was transmitted to PACS and converted into 3D reconstruction data. Then the reconstructed image was rotated to obtain a better observation position. All CT images were evaluated by two experienced radiologists working for more than 5 years, and the measurement reports were made after the two doctors reached a consensus.

**Fig. 1:**
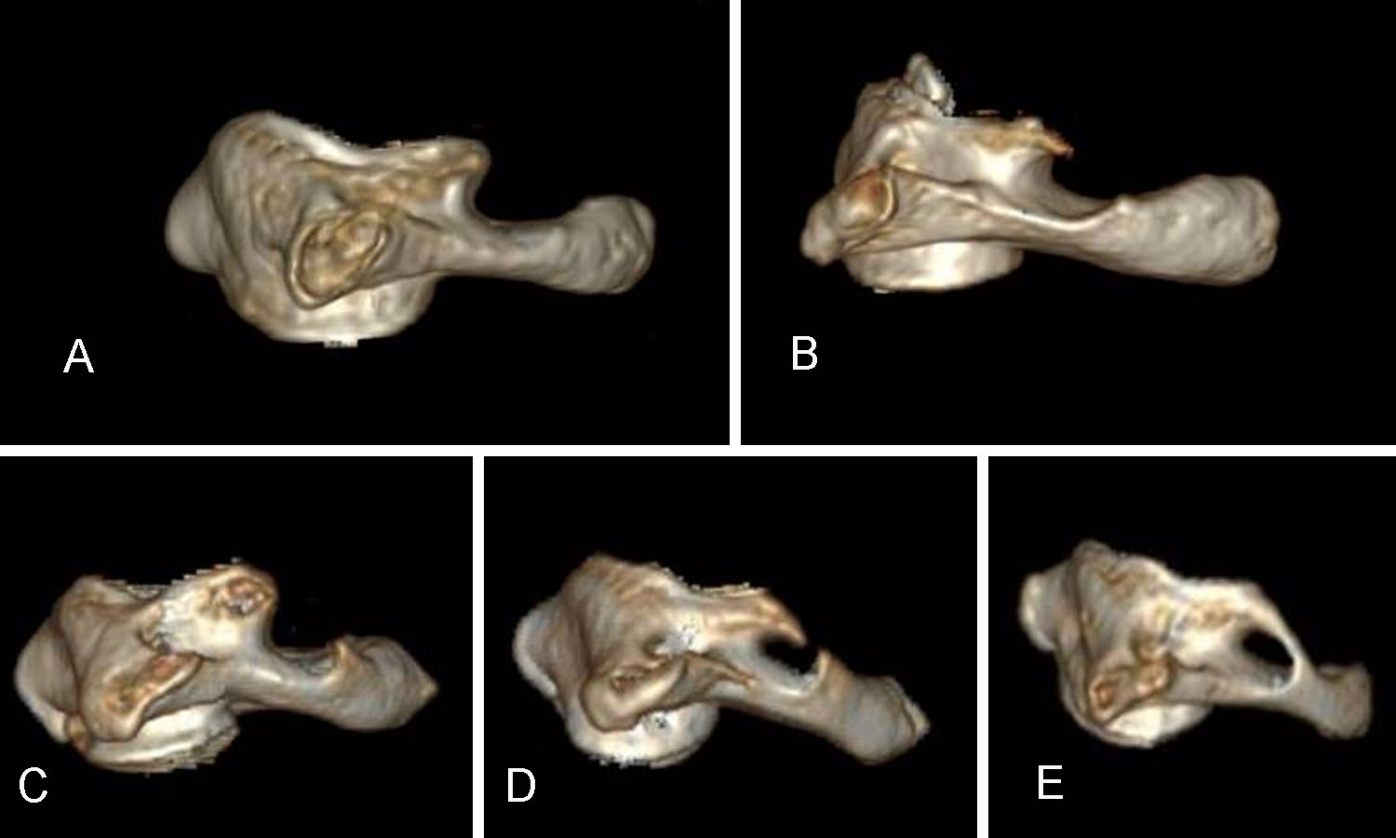
Classification map of atlas vertebral artery sulcus: **A** no process type; **B** upper process type; **C** lower process type; **D** double process type; **E** posterior ring type

Three-dimensional reconstruction data of 600 atlas vertebral artery sulci in 300 participants were completely obtained. The CT surface reconstruction images of the atlas vertebral artery sulci were classified by anatomical morphology, which were roughly divided into five types: no process type, upper process type, lower process type, double process type, and posterior ring type (Fig. 1). To assess the effect of the morphology of the atlas vertebral artery sulcus on the pedicle screw placement, a series of parameters were measured, and the details of such parameters are described as follows.

The point a was the intersection at 20 mm, next to the posterior tubercle of the atlas and 2 mm above the lower edge of the posterior arch [9], and points c and d were the outermost wall of the atlas spinal canal and the innermost wall of the vertebral foramen; the line cd was measured as the pedicle width.

The point o was the midpoint of cd, and the intersection of the extension line of the ao line and the anterior arch of the atlas was point b, so the line ab was measured as the pedicle length of the atlas. The line ao was the depth of grinding drilling for C1 pedicle screw placement.

**Fig. 2:**
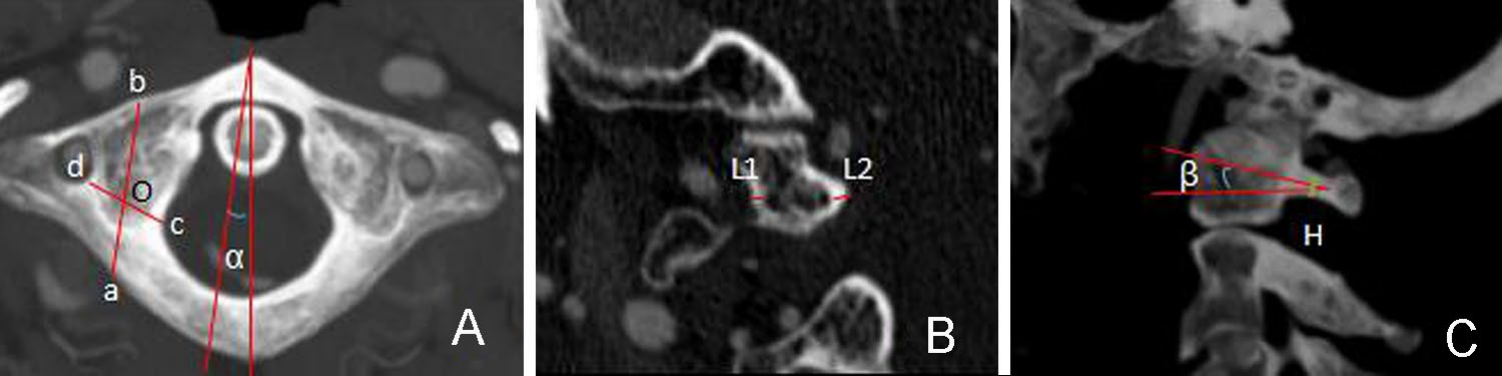
The schematic showing the parameters measured in this study. A The line ab was measured as the C1 pedicle length, and the line cd was measured as the pedicle width

The angle ∠α was measured as pedicle medial angle (Fig. 2A).

L1 and L2 were measured as the outer and inner wall thickness of the C1 pedicle on coronal section CT scans, respectively (Fig. 2B).

On coronal section CT scans, H was measured as the minimum height of the pedicle, and angle ∠β as the cephalad angle of the C1 pedicle (Fig. 2C).

One-way analysis of variance and independent sample *T* test were performed using SPSS17.0 software. One-way analysis of variance was used for statistical analysis when the variance was homogeneous, and nonparametric test was used for comparison when the variance was not homogeneous. *P* < 0.05 was set as the significant difference.

The line ao was the depth of grinding drilling for C1 pedicle screw placement, and the angle ∠α was measured as pedicle medial angle; 2B, L1 and L2 were measured as the outer and inner wall thickness of the C1 pedicle on coronal section CT scans, respectively; 2C, H was measured as the minimum height of the pedicle, and angle ∠β was measured as the cephalad angle of the C1 pedicle.

## Results

Of the 600 atlas vertebral artery sulci, CT three-dimensional reconstruction images showed that the distribution of the five types were as follows: 494 vertebral artery sulci were classified as no process type (82.33%, 494/600), 29 as upper process type (4.83%), 25 as lower process type (4.17%), 19 as double process type (3.17%), and 33 as posterior ring type (5.50%).

One-way ANOVA tests showed that the five groups differed significantly in the parameter of ao, L2, H, ∠α and ∠β. One-way ANOVA with the LSD post hoc tests showed that the parameter ao of the group of the no process type was less than that of the group of the upper or lower process type (*P* < 0.05), and ao of the group of the lower process or posterior ring type was less than that of the group of the upper type (*P* < 0.05).

One-way ANOVA with the LSD post hoc tests showed that the parameter L2 of the group of the no process type and lower process type was less than that of the group of the upper or double process type or posterior ring type (*P* < 0.05), and L2 of the group of the upper process was less than that of the group of the upper type or posterior ring type (*P* < 0.05).. One-way ANOVA with the LSD post hoc tests showed that the parameter H of the group of the no process type and upper process type was larger than that of the group of the double process type or the posterior ring type (*P* < 0.05), and the parameter ∠α of the group of the no process type was less than that of all the other four group (*P* < 0.05). In regard to ∠β, one-way ANOVA with the LSD post hoc tests showed that this parameter of the group of the no process type was less than that of all the other four group (*P* < 0.05), and the parameter of the group of the upper, lower or double process type was less than that of the group of the posterior ring type (*P* < 0.05) (Table 1).

**Table 1:** Morphological observation of the atlas vertebral artery sulcus

Variables	No process type	Upper process type	Lower process type	Double process type	Posterior ring type	Total	F	*P*
Number of atlas vertebral artery	494	29	25	19	33	600	/	/
sulci (*N*) Proportion (%)	82.33	4.83	4.17	3.17	5.50	100	/	/
ab (mm)	25.94 ± 0.88	25.76 ± 0.77	25.85 ± 0.68	25.96 ± 0.56	26.09 ± 1.21	25.94 ± 0.88	0.582	0.676
cd (mm)	9.71 ± 0.44	9.64 ± 0.49	9.70 ± 0.43	9.74 ± 0.50	9.70 ± 0.38	9.71 ± 0.44	0.192	0.942
ao (mm)	10.17 ± 0.51^a^	10.50 ± 0.56^bd^	10.22 ± 0.48^c^	10.49 ± 0.56^b^	10.23 ± 0.39^c^	10.20 ± 0.51	4.454	0.001
L1 (mm)	1.62 ± 0.16	1.60 ± 0.15	1.63 ± 0.15	1.68 ± 0.15	1.62 ± 0.16	1.62 ± 0.16	0.792	0.531
L2 (mm)	2.45 ± 0.15^e^	3.14 ± 0.26^ fg^	2.43 ± 0.18^e^	3.24 ± 0.20^fh^	3.19 ± 0.18^f^	2.55 ± 0.30	327.959	0.000
H(mm)	4.23 ± 0.48^i^	4.32 ± 0.60^i^	4.20 ± 0.39	3.98 ± 0.45^j^	4.03 ± 0.39^j^	4.21 ± 0.48	2.686	0.031
∠α (°)	6.73 ± 0.79^ k^	9.64 ± 0.32^ m^	9.61 ± 0.28^ m^	9.64 ± 0.25^ m^	9.64 ± 0.28^ m^	7.24 ± 1.32	342.988	0.000
∠β (°)	5.20 ± 0.30^n^	7.63 ± 0.37^rs^	7.73 ± 0.37^rs^	7.81 ± 0.40^rs^	9.82 ± 0.28^rt^	5.76 ± 1.31	2462.883	0.000

F stands for the test statistic in ANOVA. a: vs. b *P* < 0.05; c: vs. d *P* < 0.05; e: vs. f *P* < 0.05; g: vs. h *P* < 0.05; L1: vs. L2 *P* < 0.05; i: vs. j*P* < 0.05; k: vs. m *P* < 0.05; n: vs. r *P* < 0.05; s: vs. t *P* < 0.05

Then, when the 600 atlas vertebral artery sulci were divided into male and female groups, independent sample *T* tests showed that the parameters ao and ∠α were significantly different between groups. The parameter of ao and ∠α of the male group were larger than that of the female group (Table 2).

**Table 2:** Observation of the atlas vertebral artery sulcus in males and females

Variables	Male	Female	t	*P*
Case number (N) ab (mm) cd (mm) ao (mm) L1 (mm) L2 (mm) H (mm) ∠α (°) ∠β (°)	312 25.98 ± 0.93 9.74 ± 0.44 10.24 ± 0.53^v^ 1.62 ± 0.16 2.54 ± 0.29 4.21 ± 0.50 7.36 ± 1.34^v^ 5.76 ± 1.33	288 25.90 ± 0.83 9.68 ± 0.44 10.15 ± 0.49^w^ 1.63 ± 0.16 2.55 ± 0.30 4.22 ± 0.46 7.12 ± 1.29^w^ 5.77 ± 1.29	/ 1.124 1.604 2.131 − 0.722 − 0.363 − 0.359 2.224 − 0.068	/ 0.261 0.109 0.033 0.470 0.717 0.720 0.027 0.946

v: vs w *P* < 0.05.T stands for the test statistic in independent sample *T* test

## Discussion

Posterior fixation using pedicle and rod system is an important surgical method for the treatment of atlantoaxial dislocation, fracture, and instability. Because of its better biomechanical properties than atlas lateral mass screw, atlas pedicle screw has been gradually applied in clinic [14]. The atlas is located at the top of the spine and has great variability and structural complexity compared with other vertebral bodies. The atlantoaxial joint is adjacent to important anatomical structures such as vertebral artery and cervical spinal cord, so the difficulty and risk of screw placement in this part were significantly increased. The vertebral artery sulcus is an important anatomical landmark for atlas pedicle. The diameter and variation of the vertebral artery sulcus directly affect the placement of pedicle screws [15–17]. Some scholars described the morphology of the atlas vertebral artery sulcus, but neither of them classified the vertebral artery sulci and investigated the effect of different types on pedicle screw placement [11, 18–20]. Therefore, these previous studies were different from the current study. There have also been studies guiding the placement of pedicle screws according to the height of the atlas pedicle, but few reports investigating the effect of vertebral artery sulcus on pedicle screw placement [21–24].

In this experiment, the atlas vertebral artery sulcus was divided into five types: no process type, upper process type, lower process type, double process type, and posterior ring type. Among them, the no process type (494, 82.33%) was the most common, and the double process type (19, 3.17%) was the least. The rate of overall abnormality was 17.67%. The length of ab in the atlas vertebral artery groove was close to 26 mm, suggesting that the screw with 24 mm length could be inserted, and cd was close to 10 mm, suggesting that the width had no significant limitation on the thickness of the pedicle screw. The thickness of the vertebral artery sulcus (H) was the key factor to determine whether pedicle screws can be inserted or the thickness of screws [23]. In this study, the pedicle heights of the no process type and the upper process type were higher than those of the double process type and the posterior ring type. The results of this study showed that in most cases, atlases with no process type and the upper process type could accommodate a screw with a diameter of 4 mm, and those of the lower process type, the double process type and the posterior ring type could accommodate a screw with a diameter of 3.5 mm. A small number of pedicle heights of the five types were less than 3.5 mm, and this part did not recommend the placement of pedicle.

When the pedicle screw was inserted, the insertion point and medial angle were important to avoid potential neurovascular injury [10]. In this study, the medial angle of the upper process type, lower process type, double process type and posterior ring type was about 9.6°, which was significantly greater than 6.7° of the no process type, and the medial angle of males was also significantly greater than that of females. Therefore, attention should be paid to the grasp of the medial angle when the pedicle screw is inserted into the atlas of the no process type and different genders. In addition, the inner side wall (L2) of the atlas pedicle was thicker than the outer wall (L1), suggesting that the screw could easily migrate to the outer side when it passed through the pedicle channel. The inner side walls of the upper process type, double process type and posterior ring type were thicker than those of the no process type and the lower process type, so that the pedicle screw was more likely to migrate to the outer side. The vertebral artery was close to the outer side wall of the pedicle, and the outward migration could easily hurt the vertebral artery, resulting in serious complications. Before pedicle screw insertion, the opening of the nail track was required. If a manual drill or ordinary electric drill is used, it can easily shift to the outer side during the opening. In the operation, our experience was that the using a high-speed grinding drill with a grinding head of 2.5 mm to open the track could effectively avoid the lateral migration of the nail track. In this study, ao represents the distance from the insertion point to the narrowest cross section of the pedicle. When a high-speed grinding drill was used to drill holes, the grinding head should be drilled at least deeper than ao to avoid screw offset. The ao of men was significantly greater than that of women and the depth needs to be increased. If the upper inclination angle of the pedicle screw was too large, the vertebral artery was easily injured [17]. In this study, the upper inclination angle of the upper process type, the lower process type and the double process type was about 7.7°, and the upper inclination angle of the posterior ring type was about 9.8°, which was significantly greater than the upper inclination angle of the no process type. 5.2°. Therefore, the upper inclination should not be too large when the pedicle screw is inserted in the no protrusion type.

### Limitation

This study also has certain limitations. First, we only collected CT data of a hospital in Luxian County, and did not collect data of atlas vertebral artery groove between different regions and races. Second, the number of included samples was limited, and there may be other types of atlas vertebral artery groove variation. Finally, only the CT data of healthy adults were analyzed, and there was no comparative study of different surgical methods corresponding to different types of atlas vertebral artery groove.

## Conclusion

The morphology of atlas vertebral artery sulcus was studied by three-dimensional CT reconstruction. Atlas vertebral artery with no process type was the most common among of the five different types, and the medial angle and cephalad angle of the atlas pedicle in this type were the smallest. When the pedicle screws are inserted, the above two angles should not be too large. Male’s ao was larger than that of female’s, and when high-speed grinding drill is used to drill the opening, the deviation of the nail track should be avoided.
